# Abrupt upwelling and CO_2_ outgassing episodes in the north-eastern Arabian Sea since mid-Holocene

**DOI:** 10.1038/s41598-022-07774-4

**Published:** 2022-03-09

**Authors:** Syed Azharuddin, Pawan Govil, Thomas B. Chalk, Mayank Shekhar, Gavin L. Foster, Ravi Mishra

**Affiliations:** 1 Birbal Sahni Institute of Palaeosciences, 53 University Road, Lucknow, U.P. 226007 India; 2 School of Ocean and Earth Science, National Oceanography Centre Southampton, University of Southampton Waterfront Campus, Southampton, SO14 3ZH UK; 3 National Centre of Polar and Ocean Research, Headland Sada, Vasco-da-Gama, Goa 403 804 India; 4 Present Address: School of Earth and Environmental Sciences, Seoul National University, Gwanak-gu, Seoul, 08826 South Korea

**Keywords:** Biogeochemistry, Carbon cycle

## Abstract

Identifying the causes and consequences of natural variations in ocean acidification and atmospheric CO_2_ due to complex earth processes has been a major challenge for climate scientists in the past few decades. Recent developments in the boron isotope (δ^11^B) based seawater pH and pCO_2_ (or pCO_2_^sw^) proxy have been pivotal in understanding the various oceanic processes involved in air-sea CO_2_ exchange. Here we present the first foraminifera-based δ^11^B record from the north-eastern Arabian Sea (NEAS) covering the mid-late Holocene (~ 8–1 ka). Our record suggests that the region was overall a moderate to strong CO_2_ sink during the last 7.7 kyr. The region behaved as a significant CO_2_ source during two short intervals around 5.5–4 ka and 2.8–2.5 ka. The decreased pH and increased CO_2_ outgassing during those abrupt episodes are associated with the increased upwelling in the area. The upwelled waters may have increased the nutrient content of the surface water through either increased supply or weaker export production. This new dataset from the coastal NEAS suggests that, as a potential result of changes in the strength of the El-Nino Southern Oscillation, the region experienced short episodes of high CO_2_ outgassing and pre-industrial ocean acidification comparable to or even greater than that experienced during the last ~ 200 years.

## Introduction

The recent rapid increase in anthropogenic CO_2_ has led to ocean acidification negatively impacting marine carbonate ecosystems^1^. The level of carbon dioxide (CO_2_) in the atmosphere has a direct role in regulating the chemistry of the ocean. The dissolution of anthropogenic CO_2_ in seawater has lowered ocean pH and carbonate ion concentrations and potentially reduced the ability of marine organisms to calcify^2^. Pre-industrial variations in atmospheric CO_2_ are believed to be strongly influenced by changes in circulation and carbon cycling in the ocean^3–5^ where the interaction between large deep ocean carbon reserves and dynamic ocean circulation plays an important role in regulating the ocean carbonate system and air-sea gas exchange of CO_2_^3,4^. The mechanism involved in this exchange of CO_2_ consists of several complex processes which alter the magnitude of oceanic sinks (mainly cold, high latitude regions) and sources (mainly warm, low latitude and tropical regions)^3,6,7^. The ocean carbonate system consists of six co-varying parameters (pH, [CO_2_], [HCO_3_^−^], [CO_3_^2−^], total alkalinity (TA) and dissolved inorganic carbon (DIC)), with the full system resolvable provided any two of these six components are known. Moreover, surface water pH and pCO_2_ are very tightly coupled in natural systems and thus knowledge of one is enough to infer the other with high confidence^8^. On a glacial-interglacial timescale, the processes influencing the concentration of CO_2_ in the atmosphere are thought to leave a fingerprint on the surface water pH^9^. Also, the pH of the surface water is the primary control of the boron isotope composition in planktonic foraminifera^10–14^. Therefore, the boron isotope signatures (δ^11^B) of planktonic foraminifera provides a good opportunity to better understand the changes in surface ocean chemistry (pH) and the mechanisms involved in regulating the ocean carbonate system before human influence and industrialization, as well as providing a way to produce reconstructions of past pH and atmospheric CO_2_^15^.

The Arabian Sea is one of the most productive basins of the world, having the capability to release around 90 TgC yr^−1^ to the atmosphere^16,17^. The monsoon-influenced lateral and vertical circulation of nutrients in the water column plays an important role in regulating the productivity of the basin^18^. The majority of the southern, western and central parts of Arabian Sea are characterised by high productivity during the summer season (during south-west monsoon or SWM), due to high riverine flux and SWM influenced upwelling^19,20^. However, the northern and north-eastern parts of Arabian Sea experience comparatively high productivity during the winter season^21,22^, this is due to the combined effect of northeast monsoon (NEM) influenced winter mixing^23–25^ as well as the nutrient-rich water advected from Bay of Bengal via the south-eastern Arabian Sea (West Indian Coastal Current or WICC).

**Figure 1:**
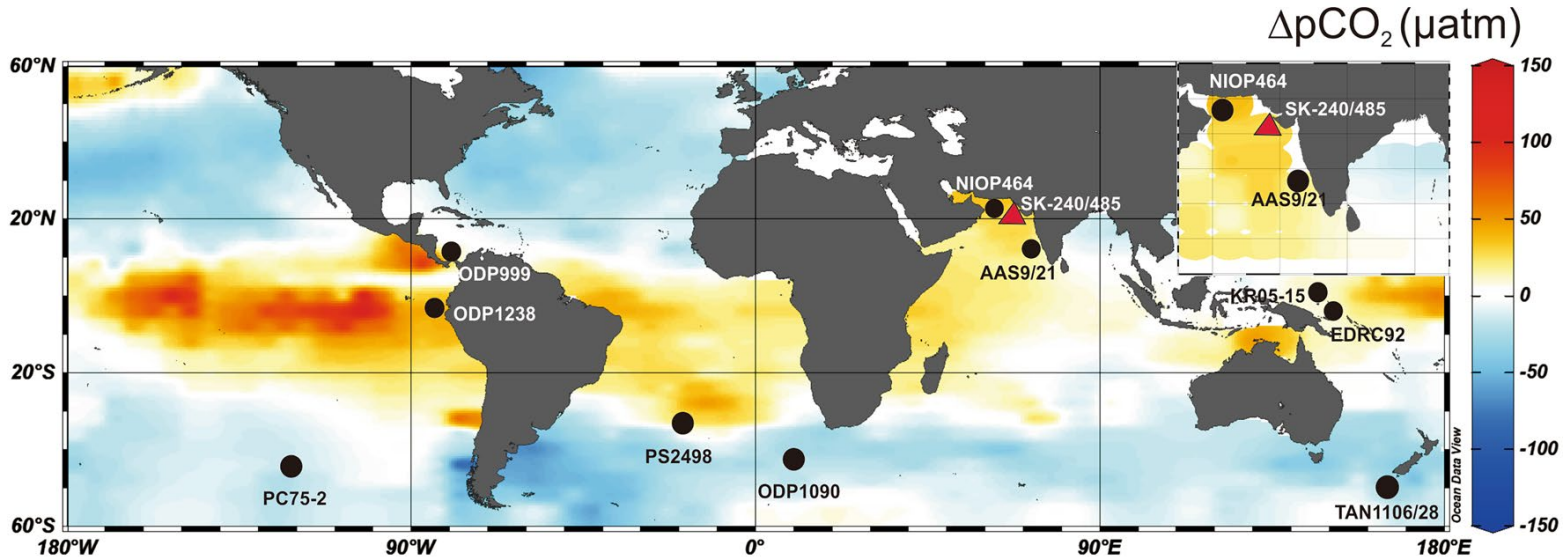
Map of modern sea surface ΔpCO_2_ distribution in the world ocean (60°N–60°S)^26^ (https://www.pmel.noaa.gov/co2/story/Surface+CO2+Flux+maps). Red triangle shows the current study site SK-240/485 nearby offshore Saurashtra, north-eastern Arabian Sea. Black circles show the other boron isotope records referred to in this study. The inset figure shows ΔpCO_2_ distribution in the northern Indian Ocean. The Map was prepared using Ocean Data View (http://www.odv.awi.de)^40^.

Surface water pCO_2_ (or pCO_2_^sw^) in the Arabian Sea is strongly influenced by physical, chemical, and biological processes which ultimately depend on the Indian Summer Monsoon and related sea surface circulation^17,26,27^. The available palaeo-pCO_2_^sw^ records from Arabian Sea suggest that the north central sector (Site NIOP464 around Murray Ridge) was a consistent source of the CO_2_ between 29 and 5 ka, whereas the south-eastern Arabian Sea (Site AAS9/21) is thought to be a significant CO_2_ sink from 23 to 5 ka^17,28^ (Fig. 1). Interestingly, both studies point towards the strength of Southwest Monsoon (SWM) as the main driver of pCO_2_^sw^ variation in the Arabian Sea in the past. Modern studies from Arabian Sea show a significant role for regional oceanography and climate on pCO_2_^sw^ variation during pre-, post-, and inter-monsoon seasons^29^. Studies from coastal regions suggest that the near shore waters tend to show much more short-term temporal and spatial pCO_2_^sw^ variations than the open ocean due to large input of terrestrial carbon and nutrients from rivers and ground waters^30,31^. More specifically, the continental shelves are CO_2_ sinks with increased pH, whereas nearshore ecosystems behave as a strong CO_2_ sources with decreased pH^30^. Studies from upwelling areas suggest that the coastal Arabian Sea, in general, has potential to behave as both a source and sink of carbon dioxide^32^. This is due to the supply of terrestrial fresh water input mixed with terrestrial detritus and nutrients (including C). On a short-term, such coastal areas show lower pH estimates than further offshore and behave as CO_2_ source. However, on the longer term, the biologically fixed carbon is deposited with the sediments in the deeper parts, which make the region a CO_2_ sink with relatively high pH^33^. Studies focused on the interaction between surface layer pCO_2_ and air-sea exchange suggest that overall the modern Arabian Sea is a weak CO_2_ source having a potential to degas ~ 460 mmol C/m^2^/yr^34^. However, studies related to the quantification of monsoon-controlled export flux to the interior parts of Arabian Sea suggests that the carbon-captured by sediments during upwelling is a potential sink of ~ 820 mmol C/m^2^/yr^35^. By virtue of such air-sea interaction, several modelling studies suggest that the inter-annual SST variability in the Indian Ocean is linked to internal oceanic cycling as well as the external atmospheric forcing^36–39^. However, these existing teleconnections are yet to be explored in past climate records.

Therefore, studying a sediment core from coastal Arabian Sea may provide new insights into the mechanism(s) of past air-sea interaction, related variations in pCO_2_^sw^ and surface water acidification, and their possible global teleconnection. Here we used the coupled analysis of δ^11^B and Mg/Ca ratio in planktonic foraminifera (*Globigerinoides ruber sensu stricto (white),* hereafter *G. ruber*) in a marine sediment core SK-240/485 collected from continental shelf offshore Saurashtra, NE Arabian Sea (Fig. 1). This study provides insights into the past pH and pCO_2_^sw^, sea surface temperature (SST) and salinity (SSS) variations to understand the role of the monsoon and associated regional oceanographic settings in regulating the past air-sea interaction in the area during mid-late Holocene.

## Results and discussion

**Figure 2:**
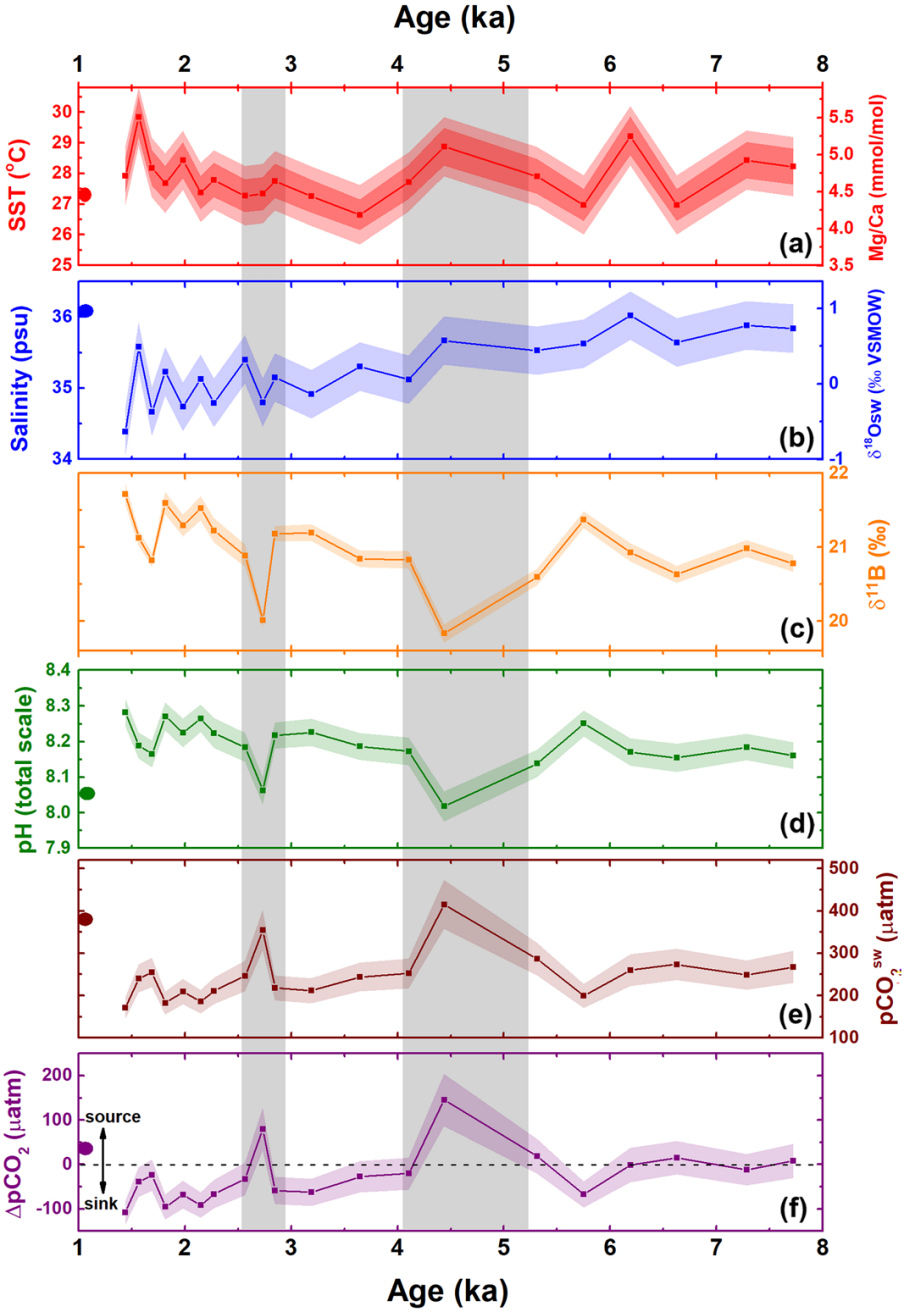
Down core variation of (**a**) Sea surface temperature (SST) (°C) and Mg/Ca ratio in *G. ruber* (mmol/mol) (**b**) Salinity (psu) and δ^18^O_sw_ (‰ VSMOW) (**c**) δ^11^B (‰) (**d**) pH (total scale) (**e**) pCO_2_^sw^ (µatm) and (**f**) ΔpCO_2_ (pCO_2_^sw^–pCO_2_^atm^) (µatm) in the core SK-240/485. Point at y-axis (left side) represents the modern (2000 AD) value around the core site. Shaded region around the curve represents 2SD error of the data. In panel (**a**) dark shade = 2SD error of Mg/Ca and light shade = 2SD error of SST. Vertical grey bands mark the abrupt CO_2_ outgassing episodes.

The surface water above core SK-240/485, is strongly influenced by the SWM with very little contribution from the NEM^41,42^, despite being located in the NE Arabian Sea. The SWM is considered as the major regulator of SST and SSS, particularly in coastal regions. This can also be observed in the monthly salinity record of nearby location (21.5°N; 68.5°E) which shows an extreme SSS decrease around October (post-SWM) (Supplementary Fig. 1). The surface-dwelling foraminifera *G. ruber* inhabits the upper water column (down to ~ 25 m) when the surface water is well stratified due to increased fresh water discharge in the basin during the SWM season. Therefore, the reconstructed signals from the *G. ruber* proxy may be biased towards the SWM season (JJAS)^43–45^. The Mg/Ca ratio of *G. ruber* at SK-240/285 shows an average value of 4.70 mmol/mol (n = 20) which corresponds to an average SST of 27.9°C in the area during mid-late Holocene (7.72–1.44 ka) (Fig. 2a). At present, the average annual SST in the region is 27.2°C with maximum (29.3°C) and minimum (24.3°C) recorded in June and February respectively (Supplementary Fig. 1). The SST recorded by core SK-240/485 ranges between a minimum of 26.7°C (around 3.65 ka) to a maximum of 29.7°C (around 1.56 ka), which shows an overall 3°C variation of SST during the last 7.7 kyr (Fig. 2a). Mg/Ca based SST records from other parts of Arabian Sea suggest 2–3 °C variation in SST during the Holocene^46–50^ (site specific details in Supplementary Table 1), which is similar to the alkenone-based SST records from northern Arabian Sea (offshore Pakistan) which show ~ 3°C variation in SST during the Holocene attributed to the effect of winter monsoon in the area^51,52^.

The δ^18^O_sw_ derived SSS in the core SK-240/485 varies between 36.0 and 34.4 psu and shows a freshening trend during mid-late Holocene (Fig. 2b). The δ^18^O_sw_ has been extensively used as a proxy for SWM-influenced evaporation-precipitation budget in the northern Indian Ocean^50,53^. The δ^18^O_sw_ at our site varies between +0.93 and −0.70 ‰ (VSMOW), with most negative value observed during the late Holocene (after ~ 4 ka; Fig. 2b and Supplementary Fig. 2b). Such a lowering of δ^18^O_sw_ suggests a significant decrease in the evaporation-precipitation budget of the area, perhaps due to an overall intensification of the SMW during 8–1 ka. A Mann–Kendall trend test reveals a significant increasing trend in δ^18^O_sw_ over our study interval [Ʈ = 0.686; *p* value (two tailed) < 0.0001] (Supplementary Fig. 2b). These observations thus point towards the increased intensification of SWM between 8 and 1 ka, as the major contribution of fresh water to the core site comes from the Indus River and small seasonal tributaries during the SWM. Previously, a δ^18^O_*G.ruber*_ based statistical and spectral study from the same site (SK-240/485) also suggested an intensifying SWM trend during the Holocene^41,42^. In addition, it is in good agreement with other studies from eastern Arabian Sea during this interval^54,55^.

The boron isotopic composition of the foraminifera (δ^11^B) shows an average value of 21.0‰ and ranges between 21.7‰ (at around 1.44 ka) and 19.8‰ (at around 4.44 ka; Fig. 2c). The reconstructed pH in the core SK-240/485 has an average value of 8.19 pH units for last ~ 7.7 kyr (Fig. 2d), which shows a good agreement with the existing pH records of the Holocene from the Pacific (~ 8.15 pH units), Atlantic (~ 8.16 pH units), and Indian (~ 8.22 pH units) Oceans^9,27,28,56–58^. More specifically, the variation of pH in the core SK-240/485 ranges between 8.02 (around 4.44 ka) and 8.28 (around 1.44 ka; Fig. 2d). The Mann Kendall trend test suggests a significant increasing trend [Ʈ = 0.386; p-value (two tailed) < 0.028] in surface water pH since 7.7 ka (Supplementary Fig. 2a). The calculated average pCO_2_^sw^ at the study site for last 7.7 kyr is 247 µatm which is ~ 22 µatm (or ppm) lower than the average atmospheric CO_2_ value (around 269 ppm) obtained from EPICA Dome-C ice core record (pCO_2_^atm^) over the same period^59^. The pCO_2_^sw^ attains a maximum of 415 µatm at around 4.4 ka and minimum of 170 µatm at around 1.4 ka (Fig. 2e). While the average pCO_2_^sw^ at the site SK-240/485 is considerably lower than the average Holocene pCO_2_^sw^ values recorded in Atlantic and Pacific Ocean^9,58,60^, it is significantly higher (~ 30 µatm) than that recorded at site AAS9/21 in the south-eastern Arabian Sea^28^ since ~ 8 ka (247 µatm at SK-240/485 vs ~ 214 µatm at AAS9/21). A modelling-based study of the evolution of pCO_2_^sw^ from the Arabian Sea suggests the existence of a strong north–south gradient in the Arabian Sea due to the seasonal sea surface circulation dynamics^17^. The south-eastern Arabian Sea shows comparatively lower pCO_2_^sw^ due to the fresh water input from the Bay of Bengal through the West Indian Coastal Current (WICC) during the NEM^41^. On the contrary, the NE Arabian Sea (including the present site) does not receive fresh water input from the WICC and therefore shows comparatively higher pCO_2_^sw^ during modern times^41,55^.

For a better understanding of the source/sink nature and quantification of air-sea CO_2_ exchange in the studied region, we calculated the ΔpCO_2_ (the difference of pCO_2_^sw^ and pCO_2_^atm^) at each time interval (Fig. 2f). The cross-plot between ΔpCO_2_ and salinity at the site SK-240/485 shows a significant positive correlation (*r* = 0.65; *p* < 0.01; R squared = 0.41 with exclusion of two points of high ΔpCO_2_ (which are probably related to intense upwelling, see below, Supplementary Fig. 3). This suggests that the SWM-influenced freshwater input plays a significant role in the ΔpCO_2_ variation of the area in the absence of strong upwelling. We found that the area was an overall CO_2_ sink during the last 7.7 kyrs with average ΔpCO_2_^sw^ around − 25 µatm (ranging between 170 and 415 µatm) which is a value characteristic of shelf regions^30^. Between 7.7 and 6.1 ka, the region was in quasi-equilibrium with the atmosphere with respect to CO_2_ (ΔpCO_2_ < 10 µatm).

The record shows two abrupt periods since 7.7 ka when the region behaved as a significant CO_2_ source, ~ 5.4–4.1 ka and ~ 2.8–2.6 ka. During these periods, pCO_2_^sw^ and ΔpCO_2_ were as high as 415 µatm and 145 µatm, respectively and surface water shows a notable 0.25 units decline in pH. During the same intervals, the δ^13^C_*G.ruber*_ (δ^13^C_GR_) from the same site (SK-240/485) shows a decrease of 0.5‰ centred around 4.5 kyr^41^ (Supplementary Fig. 4). Hence, the excess CO_2_ in the surface water we see at site SK-240/485 around ~ 4.4 ka may be a result of weaker export production and/or enhanced nutrient-rich water masses reaching to the sea surface (e.g. upwelling). A negative excursion in δ^13^C_*G.ruber*_ associated with the increased pCO_2_^sw^ due to increased upwelling of nutrient-rich water has also been observed in the south-eastern Arabian Sea during the Bølling–Ållerød (warm period)^28^. A similar pace of pH decrease along with high amounts of CO_2_ outgassing was also observed around site MD01-2416 in sub-polar North Pacific during the Bølling–Ållerød which shows the significant response of local upwelling on the pCO_2_ outgassing^61^. Based on these previous findings, we suggest that the outgassing at ~ 4.4 ka at SK-240/485 may be due to the supply of respired photosynthetic products to the surface along with the upwelled water, which increased the DIC/alkalinity ratio and thereby lowered pH and increased surface water CO_2_^61^. However, the increase in Mg/Ca SST at the same time may either be related to the near-surface habitat of *G. ruber* and upper water column stratification, rather than upwelling to the surface; the replacement of surface water currents from a lower pH and warmer region; or the influence of non-thermal effects on Mg/Ca^62^. This latter possibility is discussed in more detail in the next section.

### Non-thermal influences on foraminiferal Mg/Ca

**Figure 3:**
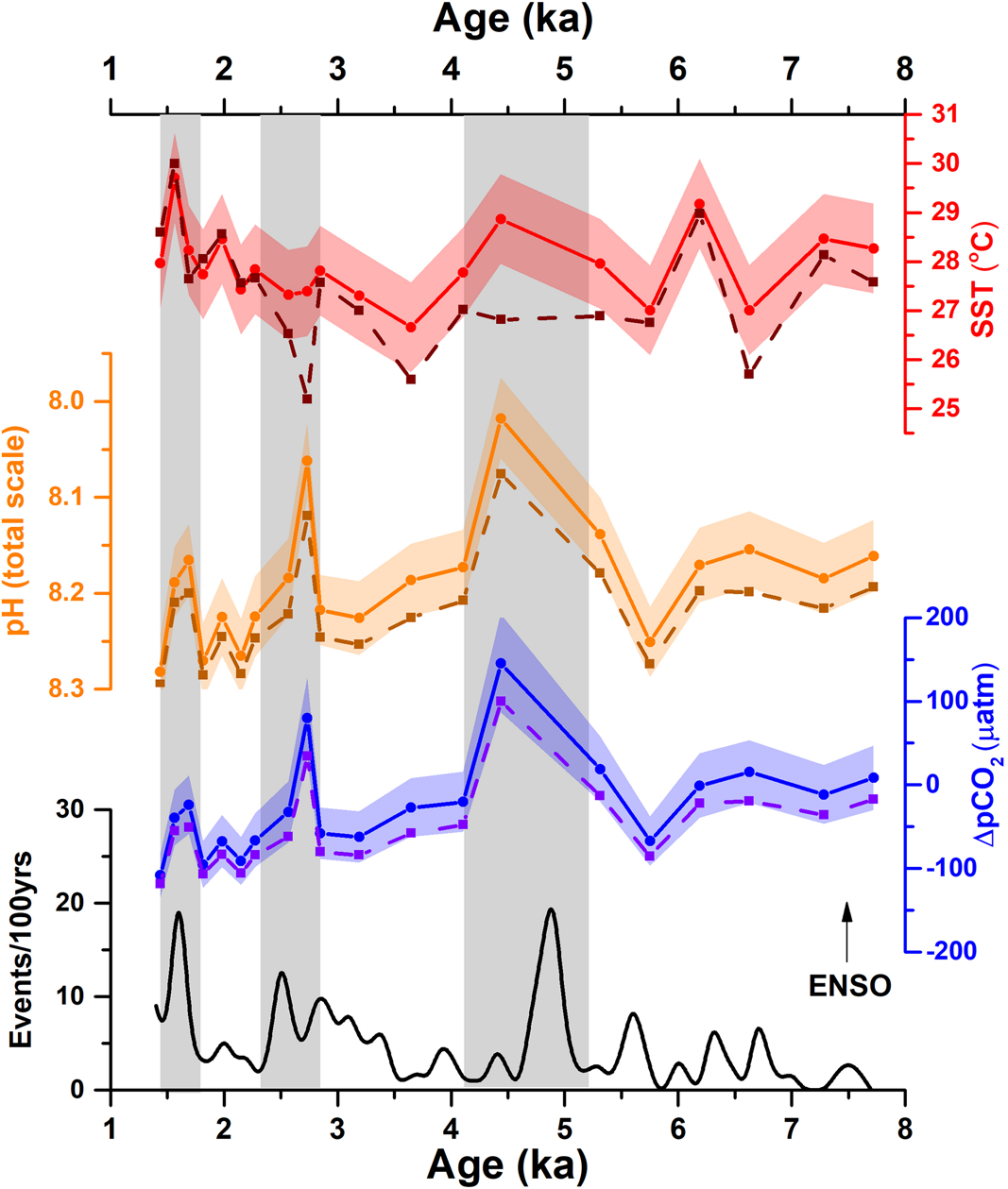
Comparison of original (solid lines) SST, pH and ΔpCO_2_ and nonthermal influence adjusted data (dashed lines) of site SK-240/485 with the frequency of ENSO events per 100 year^66^. Shaded region around the curve represents 2SD error of the data. Vertical grey bands mark the abrupt variation in the proxy record.

Several studies suggest that the foraminifera bound Mg/Ca may be impacted by other environmental variables than temperature known as non-thermal effects^62^, including salinity^63^ or carbonate system parameters^61,64,65^. These studies highlight that the local changes in seawater pH may exert a significant bias on the planktonic foraminifera Mg/Ca which lessens its correspondence to sea surface temperature (SST)^61,65^. Based on such observations, a correction protocol has been suggested for the non-thermal influence on planktonic foraminifera Mg/Ca using MgCarb package^62^. We compared the original data with MgCarb-adjusted results to check the effect of such influences in our Arabian Sea record (Fig. 3). As expected, for much of the record, the MgCarb-adjustment causes little variation. However, a difference in calculated SST of ~ 2°C is observed at 4.4 and 2.7 ka (Fig. 3) where the lowest pH is observed. Because SST has a more minor control on the calculated pH, the adjusted pH record is largely within error of the original record but with a difference of ~ 0.06 units during 4.4 and 2.7 ka, corresponding to a maximum difference of ~ 50 µatm in ΔpCO_2_ (with the adjusted pCO2 values always being lower). Nevertheless, this adjustment represents only a minor change in the overall pH and ΔpCO_2_ trends and hence the main inferences of our results remain unchanged (Fig. 3). Additionally, the adjusted SST during the periods of abrupt drop in pH and high CO_2_ outgassing (marked by dark grey bands in Fig. 3) further supports the increased upwelling in the area at 2.7 ka.

### Teleconnection of Arabian Sea upwelling with the El-Nino Southern Oscillation

**Figure 4:**
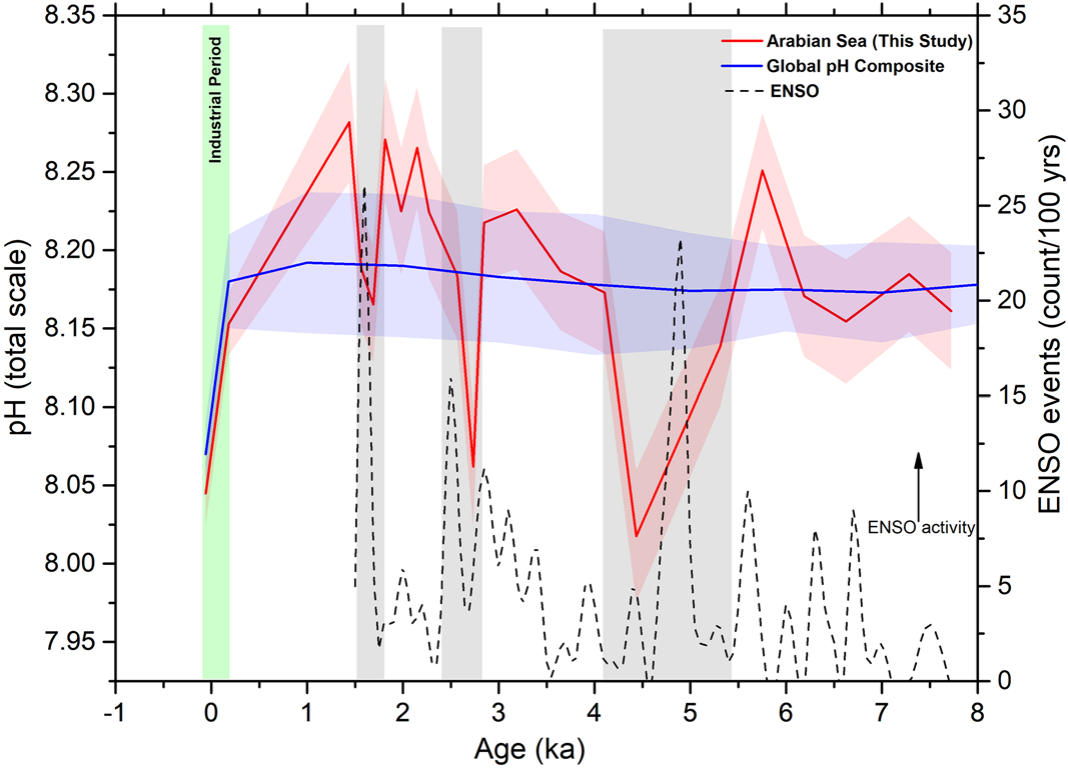
Comparison of Arabian Sea pH record around site SK-240/485 with the global pH composite^69^ and ENSO events frequency/100 yr^66^ since 8 ka. Vertical green band marks the industrial period. The modern (2010 AD) and pre-industrial (1770 AD) pH values (global and around site SK-240/485) are obtained from global ocean pH dataset^70^. Shaded regions around the curve marks the uncertainty of the dataset. Vertical grey bands mark the abrupt drop in Arabian Sea pH during high ENSO activity.

The upwelling of sub-surface waters plays an important role in controlling the CO_2_ saturation of the ocean surface. During intense upwelling, the surface waters are higher in CO_2_ saturation relative to the atmospheric CO_2_ level, decreasing surface water pH and causing CO_2_ to outgas. The modern Arabian Sea experiences intense upwelling due to the action of southwest monsoon winds over the sea surface through Ekman pumping^67^. Moreover, recent studies suggest a link between SWM and related upwelling in the Arabian Sea with the El Niño Southern Oscillation (ENSO)^68^. In this context, we compared our record of pH and ΔpCO_2_ from site SK-240/485 with the proxy record of long-term ENSO activity during the Holocene (Figs. 3, 4): the variation of sedimentation around Laguna Palcacocha, South Ecuador that is thought to reflect the variation of ENSO activity in the area^66^. The comparison reveals that the abrupt spikes in SST, ΔpCO_2_ and pH at 4.4 and 2.7 ka coincide with periods with high ENSO activity (Figs. 3, 4).

The ENSO is an important phenomenon of the tropical climate system. During positive ENSO phases basin-wide warming occurs throughout the tropical Indian Ocean^68^, with several studies suggesting that SST variations in the Indian Ocean have a strong correlation with the ENSO variability^71–74^. The Indian Ocean Basin Mode (IOBM) is the primary mode of variation of SST which has a strong impact on ENSO-induced heat-flux anomalies around Equatorial and northern Indian Ocean (including Arabian Sea)^68^. During frequent ENSO events, the IOBM shows warming causing an increase in precipitation over tropical Indian Ocean and further strengthening the South Asian High^68^. These climatic features influence the rising southwest monsoon winds over the Arabian Sea and result in the intensification of the Findlater Jet (FLJ)^75^. In turn, the FLJ transports the moisture from the southern Indian Ocean to the Indian landmass through the Arabian Sea and inter-annual variation of FLJ has been linked to the strength of El Niño and La Niña episodes^76^. The northward advancement of FLJ is reported to induce upwelling in the Arabian Sea^76–78^ and during an El Niño event the intensified FLJ advances towards the northern Arabian Sea where it induces strong coastal upwelling in the Arabian Sea via coastal Kelvin waves^68,77,79–81^. These existing teleconnections are yet to be explored in records of past climate and are difficult to test, however, our observations suggest a strong influence of ENSO events on the upwelling around the NE Arabian Sea during mid-late Holocene, possibly similar to the modern-day relationship via coastal Kelvin waves. We therefore propose such a mechanism may have induced strong upwelling around the study area during the periods of high ENSO activity, increasing ΔpCO_2_ and surface water pH.

### Comparison of global pre-industrial records with the modern analogue

**Figure 5:**
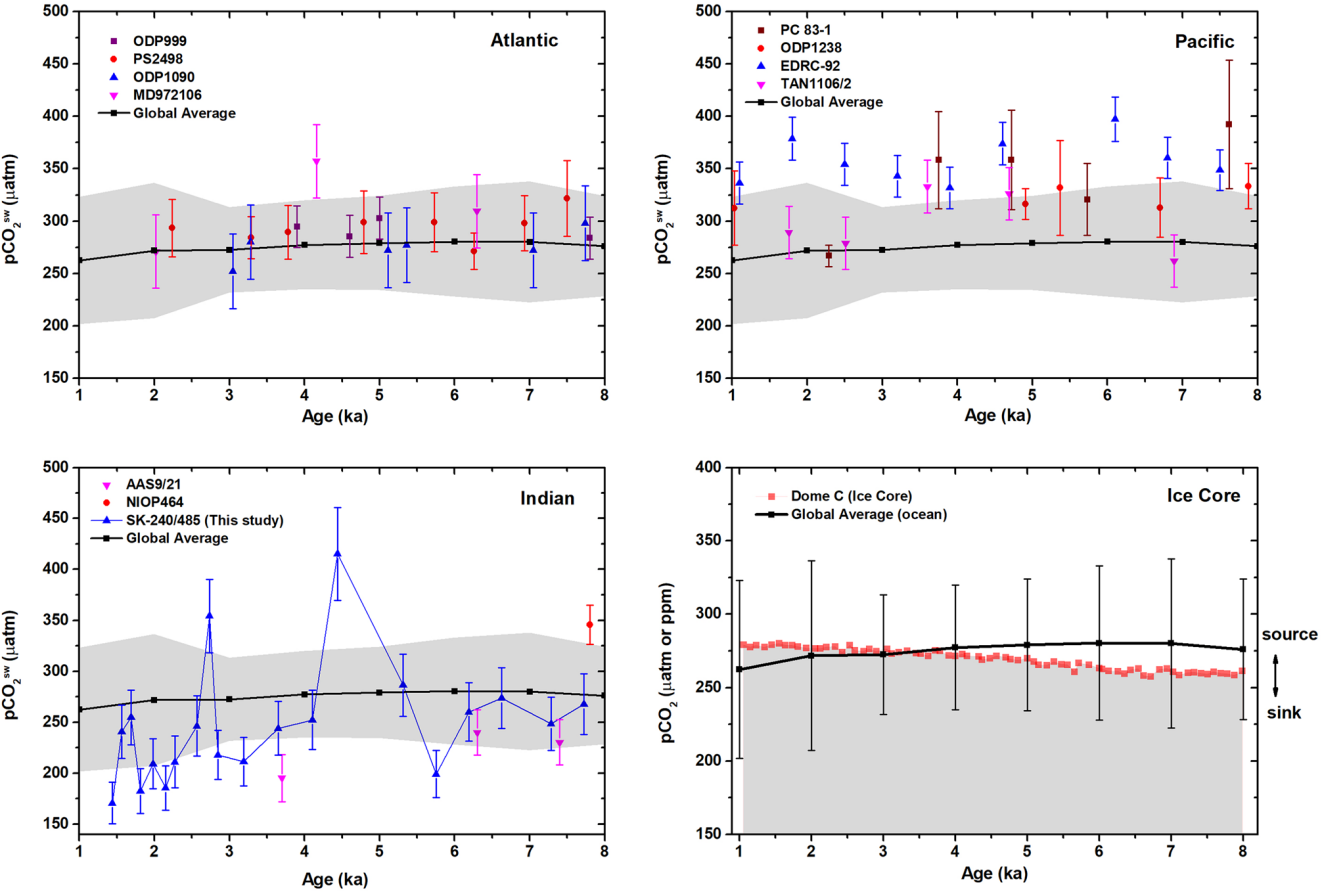
Comparison of published pCO_2_^sw^ results from Atlantic, Pacific and Indian Oceans and Dome C Ice Core with the global pCO_2_^sw^ composite^69^ during 8–1 ka. Gray shaded in Atlantic, Pacific and Indian Ocean panels mark the uncertainty of global pCO_2_ composite. Gray shaded region in the Ice Core panel indicates the sink. Refer text for more details.

For a better insight of our results in a global perspective, we have compared our pCO_2_^sw^ record with the existing records from Arabian Sea and other parts of the world ocean over the last 8–1 ka (Fig. 5 and Supplementary Table 2). We have also compared our results with global pCO_2_/pH composite curves prepared by considering several δ^11^B based pH and pCO_2_^sw^ records to understand the behaviour of north-eastern Arabian Sea in context of global source/sink patterns^69^ (See Supplementary Table 2 for more details). The pCO_2_ records from equatorial and southwest Pacific Ocean (Sites ODP1238, EDRC-92 and PC 83-1) suggest that these areas were significant sources of CO_2_ outgassing to the atmosphere during 8–1 ka^58,60,69^. In contrast, a recent study from western equatorial Pacific region (Site KR05-15) indicates that the area is a modest CO_2_ sink since the last glacial period^82^, whereas the eastern equatorial Pacific has continued to be a significant CO_2_ source, the equatorial-western Pacific is currently in near equilibrium (pCO_2_^sw^≈ pCO_2_^atm^) and the south-western Pacific has changed to a modest sink of atmospheric CO_2_ (Fig. 1)^83^. The pCO_2_^sw^ record from the ODP Site 999 in Caribbean Sea (Atlantic) indicates that the region was in near equilibrium (pCO_2_^sw^ ≈ pCO_2_^atm^) over the last 8 kyr and continues to remain this way to the present^9^. On the other hand, the site PS2498 in the south of Atlantic (~ Southern Ocean) appears as a larger CO_2_ source ~ 8 ka and is currently a modest CO_2_ sink during modern times^58^. A recent study from site TAN1106/28 around offshore New Zealand in the sub-Antarctic Pacific shows high pCO_2_^sw^ leading to the CO_2_ outgassing from the region around 4 ka^84^ and a similar increase in CO_2_ outgassing potentially caused by enhanced upwelling of CO_2_-rich deep waters around 4.5 ka, this has also been reported from site MD972106 in Atlantic sector of Sub Antarctic Zone^85^. The change in the Southern Ocean from a significant CO_2_ source (during the deglacial and into the Holocene) to a significant sink in modern times has been attributed to the resumption of Antarctic upwelling and northward advection of CO_2_-rich waters via Ekman pumping^58,86^.

The currently available δ^11^B based records from Indian Ocean are restricted to the Arabian Sea^27,28^. The ΔpCO_2_ record around site AAS9/21, albeit at low resolution, indicates that the south-eastern Arabian Sea was a significant sink during last ~ 8 kyr^28^. The present study provides new information about the changes in carbonate system around the coastal NE Arabian Sea during 7.7–1.4 ka. The data shows an overall sink in the area along with the high CO_2_ outgassing episodes in the area (as discussed in the previous sections). At the present time, the average ΔpCO_2_ and pH nearby site SK-240/485 is around 37 µatm and 8.04 units respectively^26,70^. It is worth mentioning here that the recent (2010 AD) pH nearby site SK-240/485 (8.04) is 0.03 units lower than the average pH of global ocean in 2010 AD^70^ (Fig. 4 and Supplementary Fig. 5). We have compared the pre-industrial (1770 AD) and modern (2010 AD) pH at our core site based on a recent study of global pre-industrial, modern and future pH estimates^70^. The pre-industrial pH value (during 1770 AD or ~ 0.18 BP) nearby the study area is estimated to be 8.15 which was also 0.03 units lower than the average pH of the global ocean (~ 8.18) at that time^70^. During industrialisation (between 1770 AD—present) the surface water pH around the study area varied at a similar pace to the global surface water pH (i.e. dropped ~ 0.15 pH units) due to the significant increase in global atmospheric CO_2_ levels^70^. Furthermore, with the current rate of pH change, it is estimated that the Arabian Sea, similar to the global ocean, may experience around 0.2–0.3 pH units drop in pH by 2100 if no attempt is made to mitigate emissions^70^.

## Conclusion

The present δ^11^B based pH and pCO_2_^sw^ results from the site SK-240/485 in NE Arabian Sea suggest that the region overall behaved as a net CO_2_ sink during Mid-Late Holocene, which is a typical characteristic of a shelf region. In addition, the region was nearly in equilibrium with the atmosphere during 7.7–6.1 ka. Thereafter, the area behaved as an abrupt CO_2_ source during two short periods of strong ENSO activity during 5.5–4 ka (very strong CO_2_ source) and 2.8–2.5 ka (moderate CO_2_ source). The periods of abrupt increase in ΔpCO_2_ are likely associated with the enhanced upwelling of nutrient-rich waters to the surface. It is estimated that the two intense upwelling episodes (centring around 4.4 and 2.7 ka) triggered CO_2_ outgassing and preindustrial ocean acidification similar to the modern levels. Subsequently, during 2.5–1.4 ka, the region behaved as a strong CO_2_ sink probably due to the absence of upwelling. We suggest therefore that there is evidence of intense natural ocean acidification episodes in the region over the past at times of sudden increase in carbon and nutrient supply to the surface due to increased coastal upwelling. We found that the reconstructed pH from site SK-240/2485, as well as the pre-industrial and modern value, appears to be in the range of global pH variation during the Holocene (within uncertainty) except during these two abrupt periods of intense upwelling. A comparison of our data with available records of past ENSO activity suggests a possible teleconnection of NE Arabian Sea upwelling with the timing and intensity of ENSO events during the Holocene. However, this aspect in particular needs to be confirmed in future studies from other regions of the northern Indian Ocean.

## Methods

### Study area

The analysed marine sediment core (SK-240/485) was recovered from offshore Saurashtra (Lat. 21° 16′; Long. 68° 55.99′ E; 88 m water depth), NE Arabian Sea (Fig. 1). The core covers period between the Pleistocene-Holocene boundary and the Holocene^42^ (last 12.4 kyr). However, the present study is restricted to cover the period between ~ 7.7 and ~ 1.4 ka due to insufficient foraminiferal abundance towards the bottom of the core.

The Arabian Sea is the north-western extension of Indian Ocean. It spreads around the coasts of India, Pakistan, Yemen, Oman, Somalia and the Addu Atoll (Maldives). The Indus River is the primary source of freshwater and sediment deposition in the NE Arabian Sea which receives fluxes of meltwater discharge from the Himalaya as well as runoff from monsoon precipitation^87–89^. The Indus Fan is the most extensive physiographic feature of the Arabian Sea and considered to be the second-largest submarine fan in the world^89^. Apart from the primary source, minor freshwater input also comes from the Bhadar River (a seasonal river) during the SWM season. The average annual salinity around the study area today is 36.2 psu, and the average annual sea surface temperature (SST) and ΔpCO_2_ (the difference between pCO_2_^sw^ and atmosphere) are 27.1 °C and + 36.7 µatm respectively^26,90^ (Supplementary Fig. 1) with the latter having an annual cycle of ~ 18 µatm.

### Geochemical proxies for studying ocean carbonate system

We analysed Mg/Ca ratios and δ^11^B in planktonic foraminifera (*G. ruber*). The ratio of Mg to Ca (or Mg/Ca ratio) in calcite (particularly foraminifera) serves as a proxy of past seawater temperature and has been extensively used in the world oceans^62,91–96^.The boron isotopic composition, expressed as δ^11^B (Eq. 1), of foraminifera is strongly influenced by the pH of the surface water^9–11,14^. Hence, the δ^11^B of planktonic foraminiferal shells is used as a proxy to reconstruct the pH of surface seawater and further to decipher other parameters of the ocean carbonate system, most notably pCO_2_^sw^^9^.

1 $$\updelta^{11} {\text{B}}\left( \textperthousand \right) = \left( {\frac{{\frac{{^{11} {\text{B}}}}{{^{10} {\text{B}}}}\left( {{\text{sample}}} \right)}}{{\frac{{^{11} {\text{B}}}}{{^{10} {\text{B}}}}\left( {{\text{standard}}} \right)}} - 1} \right) \times 1000$$

The geochemical analyses of foraminifera (Mg/Ca ratio and δ^11^B) were carried out at the University of Southampton, UK. For determination of Mg/Ca as well as δ^11^B for each sample 170 individuals of *Globigerinoides ruber* (white variety; sensu-stricto type) with a size range of 300 to 350 µm were hand separated. The hand-separated specimens were then cleaned following Barker et al.^97^, without applying the reductive cleaning step. Briefly, a multi-step cleaning protocol was applied consisting of crushing the foraminifera shells, followed by the removal of clay, silicate and organic matter, and a weak acid leaching with final dissolution of the carbonates using 0.5 M HNO_3_ as detailed in Foster et al.^98^. A small aliquot (~ 7%) of the cleaned and dissolved sample was analyzed for Mg and Ca (and other elemental ratios) by inductively coupled plasma mass spectrometry^9^ (ICP-MS) following Henehan et al.^99^ while the remaining sample was analysed for δ^11^B using Multi collector inductive coupled plasma mass spectrometry (MC-ICP-MS) following Foster^9^. External reproducibility of the MC-ICPMS δ^11^B method (at 95% confidence, 2SD) is based upon repeat measurements of an in house carbonate standard and is a function of sample concentration this ranges from 0.4‰ to 0.2‰ for the samples presented here^57,98,100^. Analytical precision for elemental ratios is determined at 95% confidence by the reproducibility of several in house standards and is < 5% for all elements. Strict criteria are used to rule out clay contaminated results (samples with Al/Ca > 100 μmol/mol removed). All samples are matrix matched to a gravimetrically determined standard, to eliminate Ca concentration effects on the ratios produced^99^.

The measured Mg/Ca (mmol/mol) ratio were converted to SST (°C) by using the calibration equation^101^

2 $$SST = \left( {\frac{1}{0.09}} \right)*ln\left( {\frac{Mg/Ca}{{0.38}}} \right)$$

Further, δ^18^O of seawater (δ^18^O_SW_) was calculated by using the SST derived from Mg/Ca to deconvolve ice volume effect values^95^ from δ^18^O of *G. ruber* and following equation of Bemis et al.^102^

3 $$\updelta^{18} {\text{O}}_{{{\text{sw}}}} = 0.27 + \left( {\frac{{{\text{T}}{-}16.5 + 4.8*\updelta^{18} {\text{O}}_{{{\text{calcite}}}} }}{4.8}} \right)$$

The Sea Surface Salinity (SSS) was then calculated by using δ^18^O _SW_-salinity relation for Arabian Sea suggested in equation^103^.

4 $$Salinity = \left( {\frac{{\updelta^{18} {\text{O}}_{{{\text{sw}}}} + 20}}{0.57}} \right)$$

The pH was calculated from the analysed δ^11^B values using the species-specific calibration for *G. ruber* (300–350 µm size range) of Henehan et al.^57^ (See Eqs. 1, 5).

5 $${\text{pH}} = {\text{pK}}_{{\text{B}}}^{*} - \log \left( {\frac{{ - \updelta^{11} {\text{B}}_{{{\text{sw}}}} - \left( {\updelta^{11} {\text{B}}_{{{\text{borate}}}} } \right)}}{{\updelta^{11} {\text{B}}_{{{\text{sw}}}} -^{11 - 10} {\text{K}}_{{\text{B}}} \left( {\updelta^{11} {\text{B}}_{{{\text{borate}}}} } \right) - 1000 \left( {^{11 - 10} {\text{K}}_{{\text{B}}} - 1} \right)}}} \right)$$

where pK^*^_B_ is the dissociation constant for boric acid dissociation in the in-situ (temperature, salinity and pressure) conditions and calculated as per Dickson^104^, δ^11^B_sw_ is the isotopic composition of seawater having value 39.61‰^105^. ^11-10^KB is the equilibrium constant for the two existing forms of boron in seawater i.e. borate and boric acid^106^ having a value of 1.0272 ± 0.0006^107^. Since the borate ion is predominantly incorporated into the foraminifera calcite^9^, the δ^11^${\text{B}}_{{{\text{CaCO}}_{3} }}$ can be used to obtain the pH with the species and size specific (*G. ruber*) coefficients as estimated by Henehan et al.^57^ (See Eq. 5)

6 $$\updelta^{11} {\text{B}}_{{{\text{borate}}}} = \left( {\frac{{\updelta^{11} {\text{B}}_{{{\text{CaCO}}_{3} }} - 8.87 \pm 1.52}}{0.6 \pm 0.09}} \right).$$

Further, the pCO_2_^sw^ was calculated from pH (derived from δ^11^B), SST and SSS (derived from Mg/Ca ratio) and TA (calculated using modern day relationship between SSS and TA around the area using the Global Data Analysis Project data set)^70^.

All the original data calculations are done with the R computer programming platform^108^ (R Core Team 2014) using “seacarb” package, version 6.8^109^. Foraminifera bound Mg/Ca may be impacted by non-thermal effects^62^ (e.g. Gray and Evans 2019) including salinity^63^ or carbonate parameters^64^. We explored the influence of carbonate parameters using the parametrisation using MgCarb package as mentioned in Ref^62^. The uncertainties were calculated using Monte Carlo approach (n = 10,000) with consideration of the uncertainty in all relevant input parameters (with 95% confidence level) δ^11^B ± 0.25‰; Mg/Ca derived SST ± 1 °C; SSS ± 1psu; TA ± 50 µmol/kg.

## Supplementary Information

Supplementary Information 1.

Supplementary Information 2.
