# Supplementary material for: Abrupt upwelling and CO2 outgassing episodes in the north-eastern Arabian Sea since mid-Holocene
Source: Sci Rep. 2022 Mar 9;12:3830. doi: 10.1038/s41598-022-07774-4 (PMC8907218; doi:10.1038/s41598-022-07774-4)
Supplement: Supplementary file 2 — Supplementary Information 2. [file 41598_2022_7774_MOESM2_ESM.docx]

**Abrupt upwelling and CO_2_ outgassing episodes in the North-eastern Arabian Sea since mid-Holocene**

**Syed Azharuddin^1, 2^, Pawan Govil^1^*, Thomas B. Chalk^3^, Mayank Shekhar^1^, Gavin L. Foster^3^, Ravi Mishra^4^**

1. Birbal Sahni Institute of Palaeosciences, 53 University Road, Lucknow, U.P. – 226007, India
2. Present Address: School of Earth and Environmental Sciences, Seoul National University, Gwanak-gu, Seoul-08826, South Korea
3. School of Ocean and Earth Science, National Oceanography Centre Southampton, University of Southampton Waterfront Campus, SouthamptonSO14 3ZH, UK
4. National Centre of Polar and Ocean Research, Headland Sada, Vasco-da-Gama, Goa 403 804, India.

*Corresponding author: [**pawanali@gmail.com**](mailto:pawanali@gmail.com)**,** [**pawan_govil@bsip.res.in**](mailto:pawan_govil@bsip.res.in)

**This file contains**

Supplementary Text

Supplementary Tables (1, 2)

Supplementary Figures (1-5)

References

**Supplementary text**

***Constants and assumptions used in carbonate system calculations***

The constants K1 and K2 are from Lueker et al. (2000)^1^, total boron content was from Lee et al. (2010)^2^, P, Si and SO4 2- are considered to be 0. Pressure is set at atmospheric pressure which is an inconsequential assumption given the habitat depth of the foraminifera studied here.

The inhouse standards used for the measurements were Southampton Consistency Standard (SCS) with their foram-representative 2SD values- Mg/Ca = 4.35%, Al/Ca = 3.9%).

**Supplementary Tables**

**Supplementary Table 1.** Previous studies from northern and eastern Arabian Sea along with their suggested SSTs during the Holocene. NEAS (Northeastern Arabian Sea); SEAS (Southeastern Arabian Sea); NAS (Northern Arabian Sea)

| **Core ID** | **Location** | **Method of SST reconstruction** | **SST variation during Holocene (^o^C)** | **Reference** |
| --- | --- | --- | --- | --- |
| SK 148/21 | NEAS | Mg/Ca | 2 | *Raza et al.,*^3^ |
| SN-6 | SEAS | Mg/Ca | 2.5 (since 5 ka) | *Tiwari et al.,*^4^ |
| AAS 62/1 | SEAS (Off Malabar) | Mg/Ca | 3 | *Kessarker et al.,*^5^ |
| SK-237/GC-04 | SEAS | Mg/Ca | 1.5 | *Saraswat et al.,*^6^ |
| AAS9/21 | SEAS | Mg/Ca | 2 | *Govil and Naidu,*^7^ |
| SO90-93KL | NAS (off Pakistan) | Alkenone | 3 | *Boll et al.,* ^8^ |
| 39KG/56KA | NAS (off Pakistan) | Alkenone | 3 (since 5 ka) | *Doose-Rolinsky et al.,* ^9^ |

**Supplementary Table 2.** Details of global sites considered in the present study which are explored in other records for pCO_2_ reconstruction during the last ~8 ka. The sites marked by asterisk (*) were used in the preparation of pCO2 and pH composites^10^.

| **Core ID** | **Location** | **Modern Sink/Source** | **Reference** |
| --- | --- | --- | --- |
| ODP-999* | Caribbean Sea (N. Atlantic) | Equilibrium | *Foster*^11^ |
| PS-2498* | Southern Ocean (south of Atlantic) | Sink | *Martinez-Boti et al.,*^12^ |
| PC-83-1* | Southern Ocean (south of Pacific) | Sink | *Shao et al.,*^13^ |
| TAN1106/28 | Southern Ocean (south of Pacific) | Sink | *Shuttleworth et al.,*^14^ |
| ODP-1238* | Equatorial Eastern Pacific | Source | *Martinez-Boti et al.,*^12^ |
| EDRC-92* | Equatorial Western Pacific | Equilibrium | *Palmer and Pearson,*^15^ |
| KR05-15 | Equatorial Western Pacific | Equilibrium | *Kubota et al.,*^16^ |
| AAS9/21* | Southeastern Arabian Sea (Indian Ocean) | Sink | *Naik et al.,*^17^ |
| NIOP-464* | Northern Arabian Sea (Indian Ocean | Source | *Palmer et al.,*^18^ |
| SK-240/485 | North-eastern Arabian Sea (Indian Ocean) | Source | This Study |

.

**Supplementary Figures**

**
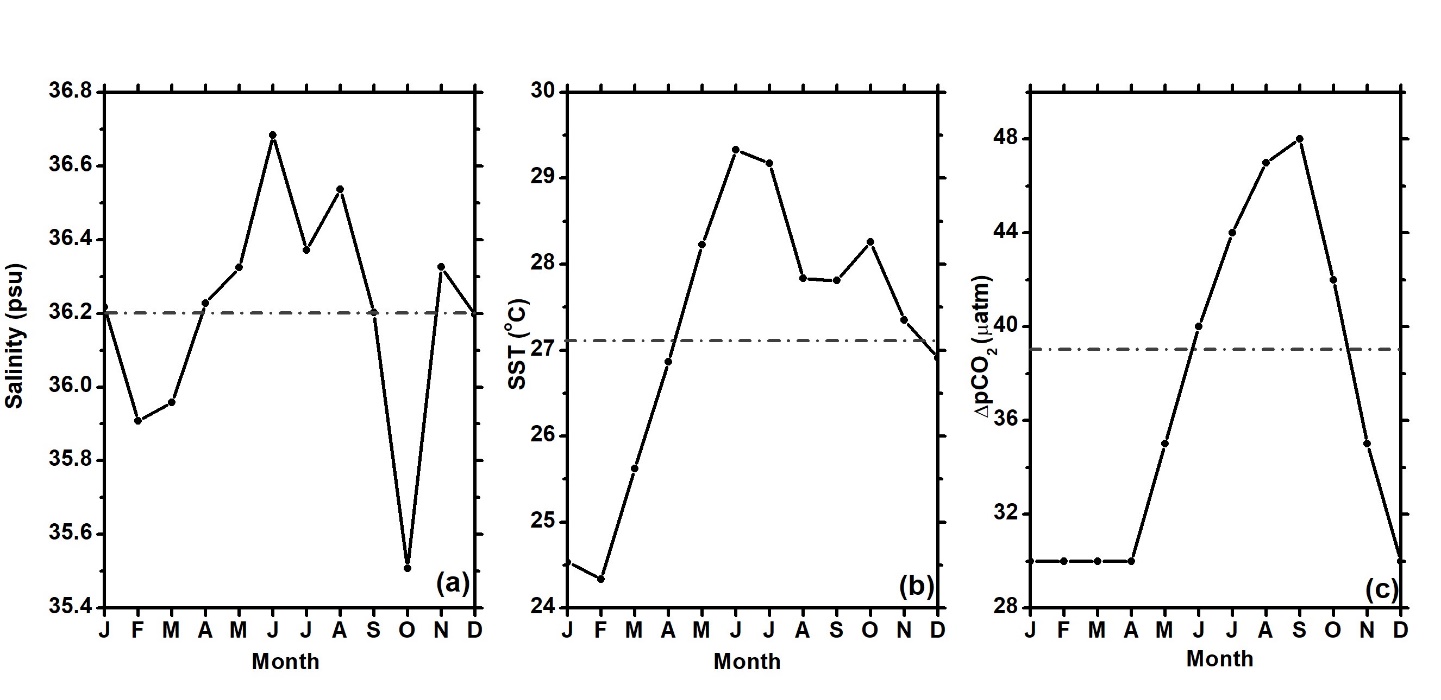
**

**Supplementary Fig. 1.** Monthly variation of (a) Salinity (b) Sea surface temperature (SST) (c) ΔpCO_2_ around the present core (21.5^o^N and 68.5^o^E)^19,20^. Dotted line represents the annual mean.

**
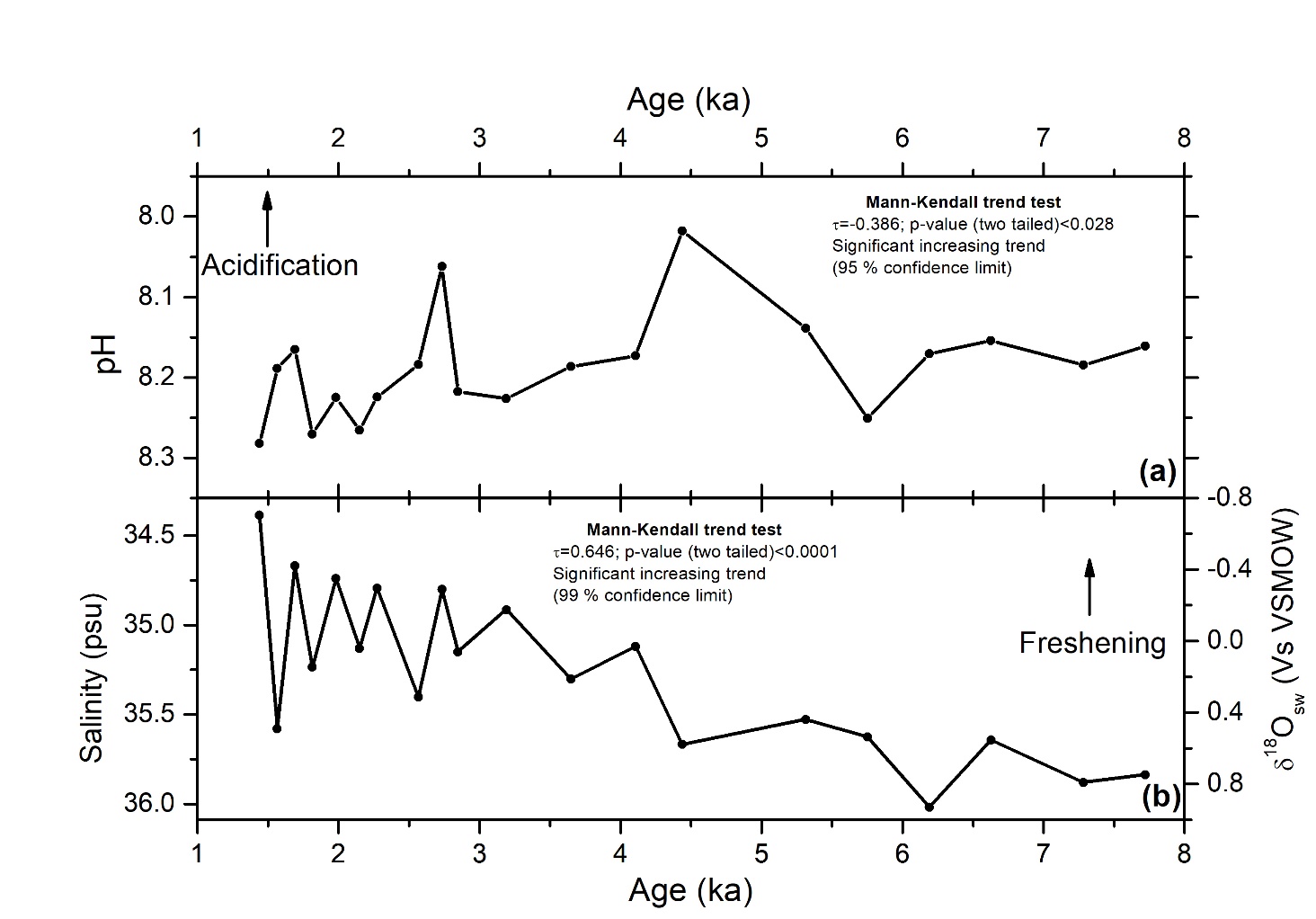
**

**Supplementary Fig. 2.** Statistical trend of (a) δ^18^Osw (‰VSMOW) and Salinity (psu) (99% confidence); (b) pH (95% confidence) in the core SK-240/485 using Mann-Kendall trend test.

**
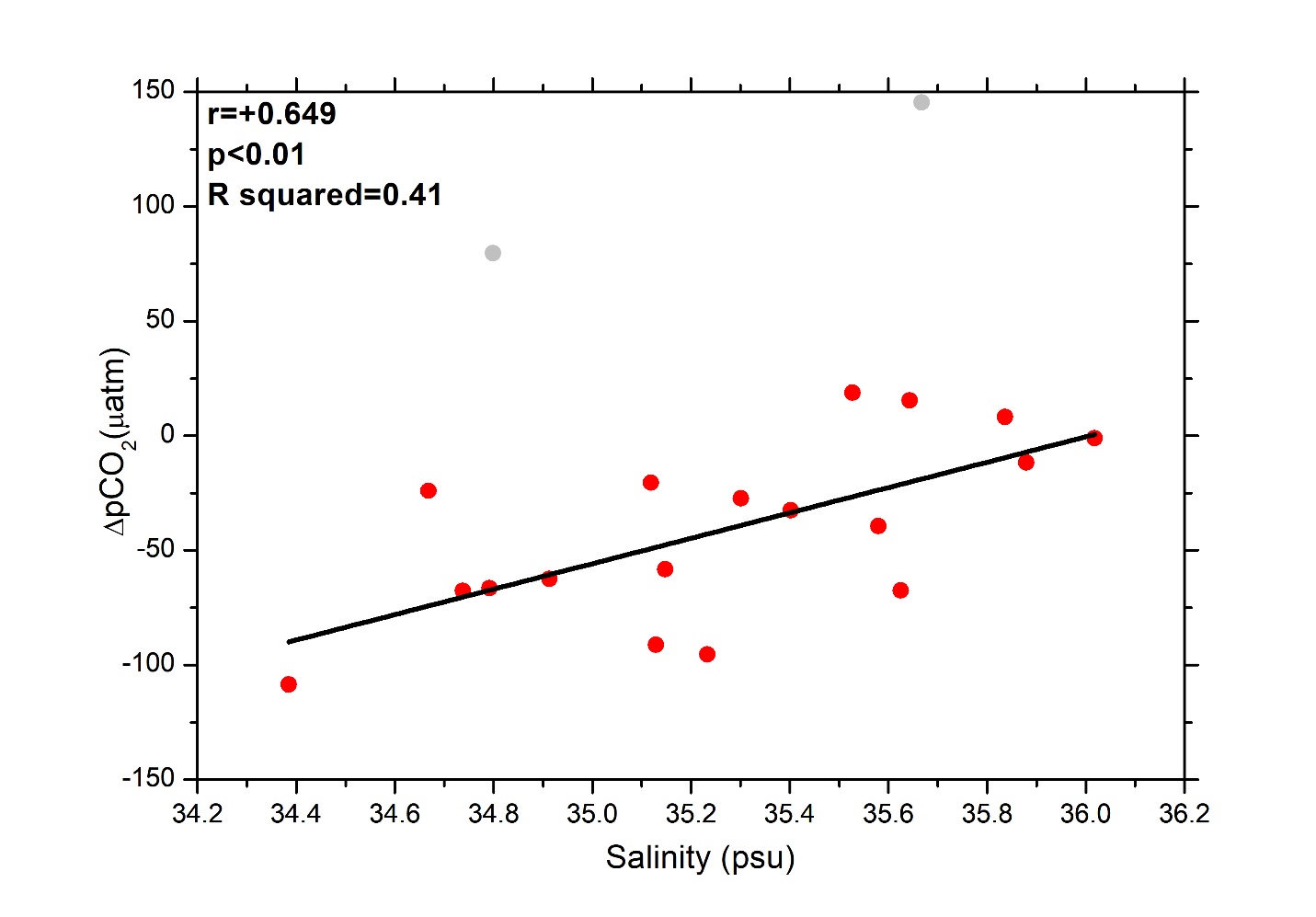
**

**Supplementary Fig. 3.** Cross-plot between ΔpCO_2_ and salinity shows significant positive correlation (excluding two points (shown in grey) of high ΔpCO_2_ values due to intense upwelling) suggesting the role of monsoon in controlling surface water pCO_2_ except during the two abrupt period of intense upwelling.


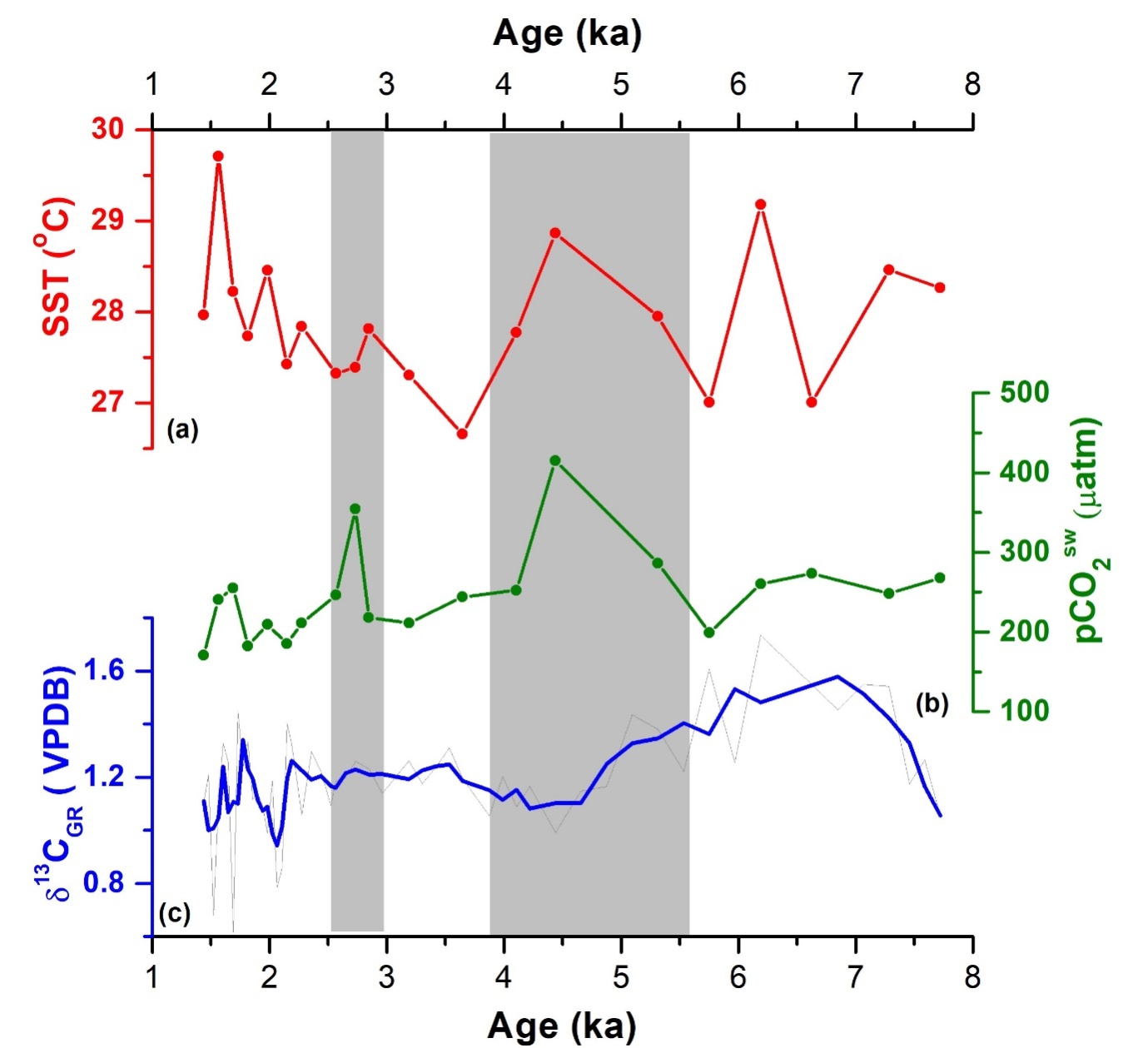


**Supplementary Fig. 4.**  Comparison of (a) SST (^o^C) (b) pCO_2_^sw^ (µatm) and (c) δ^13^C_GR_ (‰ VPDB)^21^, blue line represents 3-points averaged δ^13^C_GR_ data in the core SK-240/485. Vertical grey bands mark the abrupt CO_2_ outgassing episodes.

**
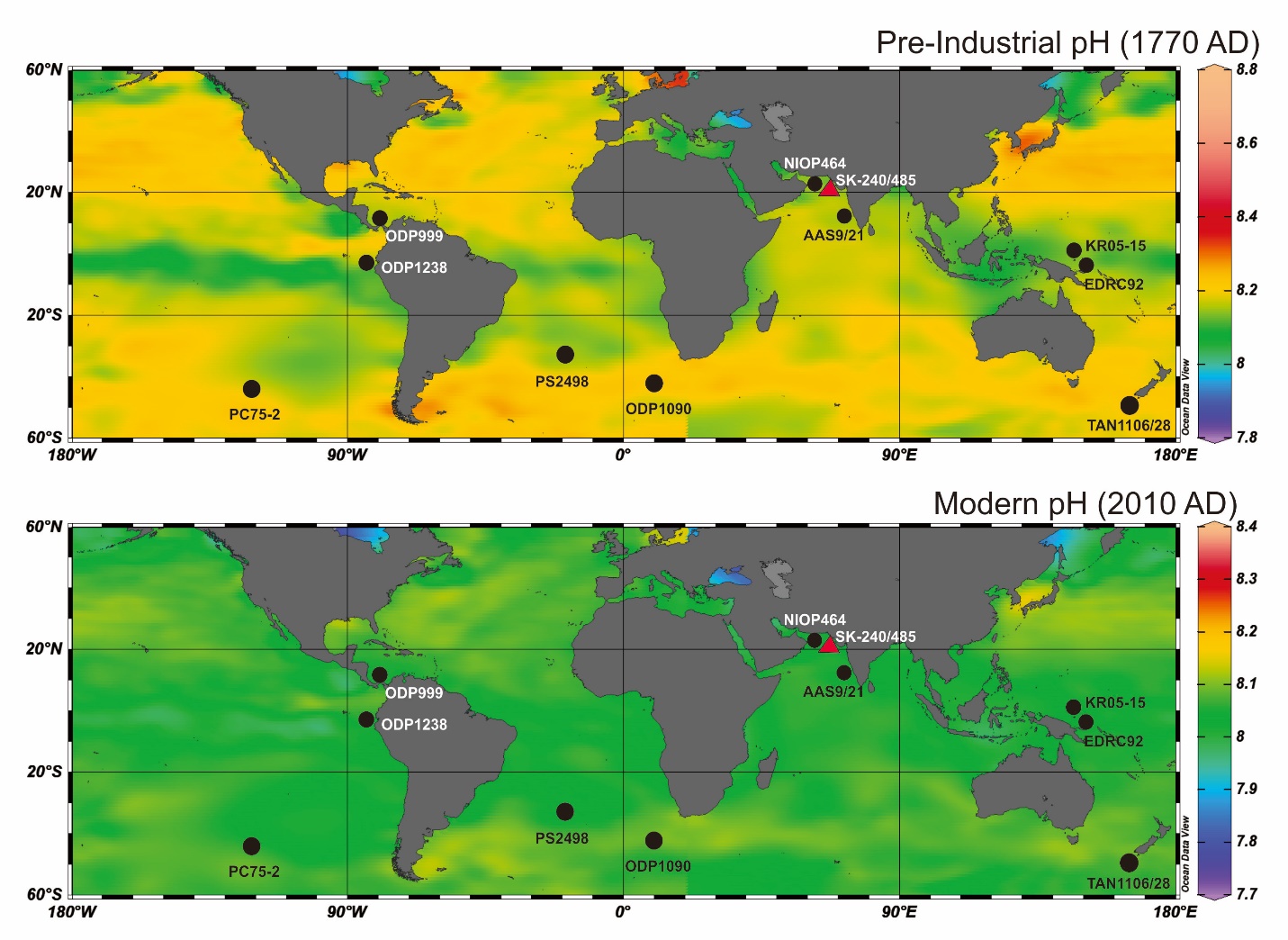
**

**Supplementary Fig. 5.** Map of sea surface pH distribution in the world ocean (60^o^N-60^o^S) during pre-industrial (1770 AD) (top) and modern (2010 AD) (bottom) times obtained from *Jiang et al.,*^22^. The modelled data product of Ref ^22^ (monthly pH, acidity and Revelle Factor from 1770 AD to 2100 AD) is freely available through NOAA/NCEI as gridded data (DOI: 10.25921/kgqr-9h49). Red triangle shows the current study site SK-240/485 nearby offshore Saurashtra, north-eastern Arabian Sea. Black circles show the other boron isotope records referred in this study. The Map was prepared using Ocean Data View (<http://odv.awi.de)23>.

**References**

1. Lueker, T. J., Dickson, A. G., & Keeling, C. D. Ocean pCO2 calculated from dissolved inorganic carbon, alkalinity, and equations for K1 and K2: validation based on laboratory measurements of CO2 in gas and seawater at equilibrium. Marine chemistry, 70(1-3), 105-119 (2000).
2. Lee, K., Kim, T. W., Byrne, R. H., Millero, F. J., Feely, R. A., & Liu, Y. M. The universal ratio of boron to chlorinity for the North Pacific and North Atlantic oceans. Geochimica et Cosmochimica Acta, 74(6), 1801-1811 (2010).
3. Raza, T., Ahmad, S. M., Steinke, S., Raza, W., Lone, M. A., Beja, S. K. & Suseela, G. Glacial to Holocene changes in sea surface temperature and seawater δ18O in the northern Indian Ocean. Palaeogeogr. Palaeoclimatol. Palaeoecol, 485, 697–705 (2017).
4. Tiwari, M., Nagoji, S. S., & Ganeshram, R. S. Multi– centennial scale SST and Indian summer monsoon precipitation variability since the mid– Holocene and its nonlinear response to solar activity. *The Holocene*, 25(9), 1415– 1424 (2015).
5. Kessarkar, P. M., Purnachadra Rao, V., Naqvi, S. W. A. & Karapurkar, S. G. Variation in the Indian summer monsoon intensity during the Bølling Ållerød and Holocene. *Paleoceanography,* 28(3), 413– 425. (2013).
6. Saraswat, R., Lea, D. W., Nigam, R., Mackensen, A., & Naik, D. K. Deglaciation in the tropical Indian Ocean driven by interplay between the regional monsoon and global teleconnections. Earth Planet Sci Lett, 375, 166– 175. (2013).
7. Govil, P., & Naidu, P. D. Evaporation‐precipitation changes in the eastern Arabian Sea for the last 68 ka: Implications on monsoon variability. *Paleoceanography,* 25(1) (2010).
8. Böll, Anna, Hartmut Schulz, Philipp Munz, Tim Rixen, Birgit Gaye, and Kay-Christian Emeis. "Contrasting sea surface temperature of summer and winter monsoon variability in the northern Arabian Sea over the last 25 ka." Palaeogeography, Palaeoclimatology, Palaeoecology 426: 10-21 (2015).
9. Doose‐Rolinski, Heidi, Ulf Rogalla, Georg Scheeder, Andreas Lückge, and Ulrich von Rad. "High‐resolution temperature and evaporation changes during the late Holocene in the northeastern Arabian Sea." Paleoceanography 16, no. 4: 358-367 (2001).
10. Shao, J., Stott, L.D., Gray, W.R., Greenop, R., Pecher, I., Neil, H.L., Coffin, R.B., Davy, B. and Rae, J.W. Atmosphere‐ocean CO2 exchange across the last deglaciation from the Boron Isotope Proxy. *Paleoceanogr Paleoclimatol*. **34**(10), 1650–1670 (2019).
11. Foster, G. L. Seawater pH, pCO2sw and [CO2− 3] variations in the Caribbean Sea over the last 130 kyr: A boron isotope and B/Ca study of planktic foraminifera. *Earth Planet. Sci. Lett*. 271(1– 4), 254– 266 (2008).
12. Martínez–Botí, M.A., Marino, G., Foster, G.L., Ziveri, P., Henehan, M.J., Rae, J.W., Mortyn, P.G. and Vance, D., Boron isotope evidence for oceanic carbon dioxide leakage during the last deglaciation. *Nature*, 518(7538), 219–222 (2015).
13. Shao, J., Stott, L.D., Gray, W.R., Greenop, R., Pecher, I., Neil, H.L., Coffin, R.B., Davy, B. and Rae, J.W., Atmosphere‐ocean CO2 exchange across the last deglaciation from the Boron Isotope Proxy. *Paleoceanogr Paleoclimatol*, 34(10), 1650– 1670. (2019).
14. Shuttleworth, R., Bostock, H.C., Chalk, T.B., Calvo, E., Jaccard, S.L., Pelejero, C., Martinez–Garcia, A., Foster, G.L. Early deglacial CO2 release from the Sub–Antarctic Atlantic and Pacific Oceans, *Earth Planet Sci Lett*, https://www.sciencedirect.com/science/article/pii/S0012821X20305938. (2020)
15. Palmer, M. R., & Pearson, P. N. A 23,000–year record of surface water pH and pCO2 in the western equatorial Pacific Ocean. *Science*, 300(5618), 480–482 (2003).
16. Kubota, K., Yokoyama, Y., Ishikawa, T., Sagawa, T., Ikehara, M., & Yamazaki, T. Equatorial Pacific seawater pCO2 variability since the last glacial period. *Scientific reports,* 9(1), 1–11 (2019).
17. Naik, S. S., Naidu, P. D., Foster, G. L., & Martínez‐Botí, M. A. Tracing the strength of the southwest monsoon using boron isotopes in the eastern Arabian Sea. *Geophys Res Lett.* 42(5), 1450– 1458. (2015).
18. Palmer, M.R., Brummer, G.J., Cooper, M.J., Elderfield, H., Greaves, M.J., Reichart, G.J., Schouten, S. and Yu, J.M. Multi–proxy reconstruction of surface water pCO2 in the northern Arabian Sea since 29 ka. *Earth Planet. Sci. Lett*, 295(1–2), 49–57 (2010).
19. Key, R.M., Kozyr, A., Sabine, C.L., Lee, K., Wanninkhof, R., Bullister, J.L., Feely, R.A., Millero, F.J., Mordy, C. and Peng, T.H. A global ocean carbon climatology: Results from Global Data Analysis Project (GLODAP). *Global Biogeochem Cy*, 18(4). (2004).
20. Takahashi, T., Sutherland, S.C., Wanninkhof, R., Sweeney, C., Feely, R.A., Chipman, D.W., Hales, B., Friederich, G., Chavez, F., Sabine, C. and Watson, A., Climatological mean and decadal change in surface ocean pCO2, and net sea–air CO2 flux over the global oceans. Deep Sea Research Part II: Topical Studies in Oceanography, **56**(8-10), pp.554-577 (2009).
21. Azharuddin, S., Govil, P., Singh, A. D., Mishra, R., Agrawal, S., Tiwari, A. K., & Kumar, K. Monsoon– influenced variations in productivity and lithogenic flux along offshore Saurashtra, NE Arabian Sea during the Holocene and Younger Dryas: A multi– proxy approach. *Palaeogeogr. Palaeoclimatol. Palaeoecol,* 483, 136–146. (2017).
22. Jiang, L. Q., Carter, B. R., Feely, R. A., Lauvset, S. K. & Olsen, A. Surface ocean pH and buffer capacity: past, present and future. *Scientific reports*, 9(1), 1–11 (2019).
23. Schlitzer, Reiner. Data analysis and visualization with Ocean Data View. *CMOS Bulletin SCMO* **43**, (1), 9-13 (2015).
